# The challenges of evidence-based prehabilitation in a real-life context for patients preparing for colorectal surgery—a cohort study and multiple case analysis

**DOI:** 10.1186/s13741-024-00481-w

**Published:** 2025-01-17

**Authors:** A. D. Talen, N. L. U. van Meeteren, J. A. Barten, I. Pereboom, W. P. Krijnen, H. Jager-Wittenaar, B. C. Bongers, G. van der Sluis

**Affiliations:** 1 Department Physiotherapy, Nij Smellinghe Hospital, Drachten, The Netherlands; 2 Research Group Healthy Ageing, Allied Health Care and Nursing, Hanze University of Applied Sciences Groningen, Groningen, The Netherlands; 3 Top Sector Life Sciences & Health (Health~Holland), Wilhelmina Van Pruisenweg 104, Den Haag, 2595 AN The Netherlands; 4 Department of Anesthesiology, Erasmus Medical Centre, Dr. Molewaterplein 40, Rotterdam, 3015 GD The Netherlands; 5 Research Group Innovation of Human Movement Care, Research Center for Healthy and Sustainable Living, HU University of Applied Sciences Utrecht, Utrecht, Netherlands; 6 Department of Rehabilitation, Physiotherapy Science and Sport, University Medical Center Utrecht, Utrecht University, Utrecht, Netherlands; 7 Department of Nutrition and Movement Sciences, NUTRIM, Institute of Nutrition and Translational Research in Metabolism, Maastricht University, Maastricht, the Netherlands; 8 Department of Surgery, NUTRIM, Institute of Nutrition and Translational Research in Metabolism, Maastricht University, Maastricht, the Netherlands; 9 Department of Gastroenterology and Hepatology, Dietetics, Radboud University Medical Center, Nijmegen, The Netherlands; 10 Faculty of Physical Education and Physiotherapy, Department Physiotherapy and Human Anatomy, Research Unit Experimental Anatomy, Vrije Universiteit Brussel, Brussels, Belgium; 11 FAITH Research Group, Groningen & Leeuwarden, The Netherlands; 12 Department of Surgery, Nij Smellinghe Hospital, Drachten, The Netherlands

**Keywords:** Preoperative exercise, Multimodal prehabilitation, Fidelity, Colorectal surgery, Evaluation study

## Abstract

**Background:**

Multimodal prehabilitation programs are effective at reducing complications after colorectal surgery in patients with a high risk of postoperative complications due to low aerobic capacity and/or malnutrition. However, high implementation fidelity is needed to achieve these effects in real-life practice. This study aimed to investigate the implementation fidelity of an evidence-based prehabilitation program in the real-life context of a Dutch regional hospital.

**Methods:**

In this observational cohort study with multiple case analyses, all patients who underwent colorectal surgery from January 2023 to June 2023 were enrolled. Patients meeting the criteria for low aerobic capacity or malnutrition were advised to participate in a prehabilitation program. According to recent scientific insights and the local care context, this program consisted of four exercise modalities and three nutrition modalities. Implementation fidelity was investigated by evaluating: (1) coverage (participation rate), (2) duration (number of days between the start of prehabilitation and surgery), (3) content (delivery of prescribed intervention modalities), and (4) frequency (attendance of sessions and compliance with prescribed parameters). An aggregated percentage of content and frequency was calculated to determine overall adherence.

**Results:**

Fifty-eight patients intended to follow the prehabilitation care pathway, of which 41 performed a preoperative risk assessment (coverage 80%). Ten patients (24%) were identified as high-risk and participated in the prehabilitation program (duration of 33–84 days). Adherence was high (84–100%) in five and moderate (72–73%) in two patients. Adherence was remarkably low (25%, 53%, 54%) in three patients who struggled to execute the prehabilitation program due to multiple physical and cognitive impairments.

**Conclusion:**

Implementation fidelity of an evidence-based multimodal prehabilitation program for high-risk patients preparing for colorectal surgery in real-life practice was moderate because adherence was high for most patients, but low for some patients. Patients with low adherence had multiple impairments, with consequences for their preparation for surgery. For healthcare professionals, it is recommended to pay attention to high-risk patients with multiple impairments and further personalize the prehabilitation program. More knowledge about identifying and treating high-risk patients is needed to provide evidence-based recommendations and to obtain higher effectiveness.

**Trial registration:**

NCT06438484.

## Introduction

Colorectal surgery is a frequently performed treatment for ulcerative colitis, Crohn’s disease, recurrent diverticulitis, and most frequently for colorectal cancer. Of the patients undergoing colorectal surgery, 25% to 73% develop postoperative complications (Mayo et al. 2011; Berkel et al. 2022). These complications affect a patient’s physical functioning and quality of life and increase healthcare costs (Khuri et al. 2005). Preoperative risk factors like older age, multiple comorbidities, and modifiable factors, such as poor nutritional status and low physical fitness, are known to increase the risk of such postoperative complications (Moran et al. 2016; Rooijen et al. 2019a).

The risk of postoperative complications can be decreased before surgery by multimodal prehabilitation. The recommended modalities are physical exercise training, nutritional support, treatment of anemia, smoking and alcohol cessation, and psychological support, personalized to the factors that need improvement (Durrand et al. 2019; Molenaar et al. 2023). Studies show that prehabilitation has positive effects, reducing complications and length of stay, particularly for patients with high risk of complications, like patients with low preoperative aerobic capacity (Rooijen et al. 2019b; Heil et al. 2022a; Cortés-Guiral et al. 2021; Carli et al. 2017; Molenaar et al. 2023; Lambert et al. 2021; Klerk et al. 2021). Therefore, it is recommended that multimodal prehabilitation programs be integrated into usual care (Berkel et al. 2022; Molenaar et al. 2023; Franssen et al. 2022; Heil et al. 2022b).

Implementation of prehabilitation in real-life practice is challenging (Molenaar et al. 2023; Heil et al. 2022b). Unfortunately, the “evidence-to-practice gap” is not unique, as demonstrated by the average implementation time of 17 years (Bussemaker and KJ, de LM. 2021; Morris et al. 2011). Many implementation studies have stated that establishing the effectiveness of innovation in a research setting does not guarantee its uptake and effectiveness in usual care (Bauer and Kirchner 2020). The transition from the usage of an intervention studied under controlled research circumstances to adoption in real-life practice is complex, with many influencing contextual factors acting as barriers and facilitators (Heil et al. 2022a; Pearson et al. 2020; Rogers et al. 2020).

Barriers to the implementation of prehabilitation in real-life practice are multidimensional. For example, the implementation of prehabilitation in real-life practice in Dutch hospitals is challenged by financial and logistical factors, such as the lack of reimbursement by insurance companies (Molenaar et al. 2023). In addition, a recent qualitative study found several patient-reported barriers, including logistic challenges, the program’s complexity, and reluctance among healthcare professionals caused by the lack of evaluation outside of research settings (Heil et al. 2022b). Therefore, as a next step, research into this real-life practice by an embedded scientist observing and scientifically evaluating implementation processes in their context is of value (Vindrola-Padros et al. 2017).

To evaluate the implementation of interventions, the concept of implementation fidelity can be used, which is defined as “the scientific degree to which an intervention is implemented as intended by the program developers” (Proctor et al. 2011). High implementation fidelity is necessary for achieving the intended outcomes of interventions (Carroll et al. 2007). To date, the implementation fidelity of prehabilitation and its influence on the outcomes are almost unknown and have not yet been reported. Consequently, the primary aim of this cohort study with case analysis was to investigate the fidelity of an evidence-based multimodal prehabilitation program for high-risk patients undergoing elective colorectal surgery as implemented in a regional hospital in the Netherlands. The secondary aim was to explore the intended outcomes (i.e., changes in preoperative aerobic capacity and preoperative nutritional status, as well as postoperative recovery) following the multimodal prehabilitation program.

## Methods

### Study design

**Fig. 1:**
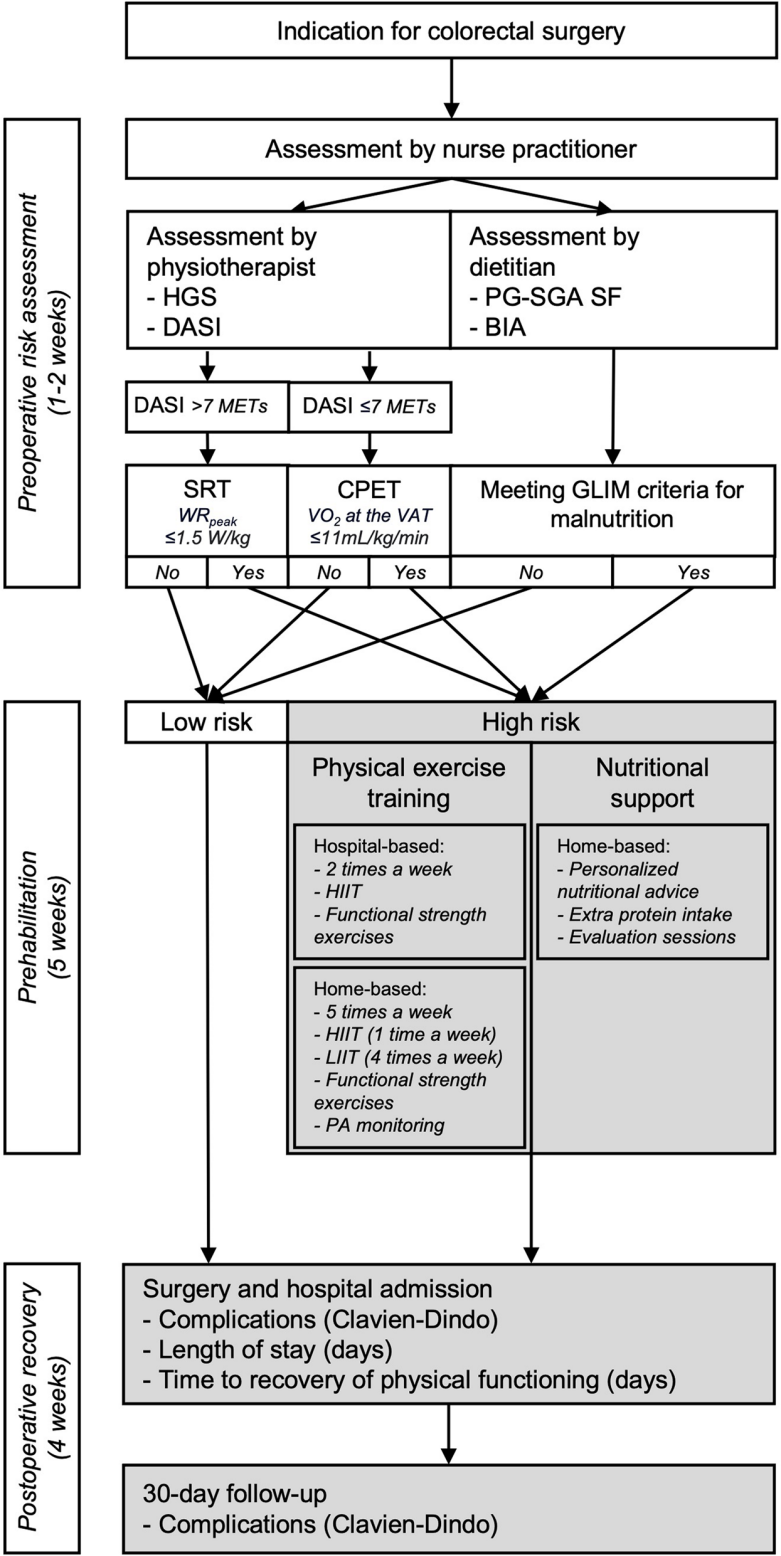
Colorectal surgery pathway of NS with the study’s focus section highlighted. Abbreviations: *BIA* bioelectrical impedance analysis, *CPET* cardiopulmonary exercise test, *DASI* Duke Activity Status Index, *GLIM* Global Leadership Initiative on Malnutrition, *HGS* handgrip strength, *HIIT* high-intensity interval training, *LIIT* low-intensity interval training, *PA* physical activity, *PG-SGA SF* Patient-Generated Subjective Global Assessment Short Form, *SRT* modified steep ramp test, *VAT* ventilatory anaerobic threshold, *VO*_*2*_ oxygen uptake, *WR*_*peak*_ work rate at peak exercise

This observational cohort study was conducted from January 2023 to June 2023 with a case analysis of the high-risk patients following the prehabilitation program in hospital Nij Smellinghe (NS). In January 2023, a multimodal prehabilitation program was implemented in the colorectal pathway, according to the concept of evidence-based medicine. Evidence-based medicine involves treating patients based on the best available clinical evidence, integrating this with individual clinical expertise, and tailoring it to the specific needs of each patient (Sackett et al. 1996). The prehabilitation program was based on the current scientific insights and guidelines, adapted to the practical possibilities and local vision of the care context of NS. This care pathway included assessing a patient’s risk of postoperative complications and offering a multimodal prehabilitation program for high-risk patients (Fig. 1). The study was approved by the Local Ethical Committee of NS (reference: 23,017/JB/AB). The STrengthening the Reporting of Observational Studies in Epidemiology (STROBE) guideline for reporting observational studies was followed (Elm et al. 2007).

The coordinating investigator, a physiotherapist, served as an embedded scientist within the colorectal pathway (Wittmayer and Schäpke 2014). This embedded researcher, affiliated with both the hospital and an academic institution, established strong relationships with healthcare professionals and collaborated closely with local teams to generate valuable insights into the implementation process. Data were extracted by the embedded researcher through desk research from patient files, complemented by direct consultations with healthcare professionals. To ensure accuracy, the physiotherapist randomly reviewed the data, and any discrepancies were resolved in consultation with an expert.

### Patients

All patients (i.e., low-risk and high-risk) of 18 years or older scheduled for elective colorectal surgery in NS were asked for their informed consent to be included in the cohort. No exclusion criteria were applied.

### Study setting

NS is a regional hospital in the Netherlands with 339 beds, where 150 patients undergo colorectal surgery annually (Sluis et al. 2015). NS is an innovative hospital in perioperative care, and the Enhanced Recovery After Surgery (ERAS) protocol has been implemented into usual care since 2018 (Greco et al. 2014). For the period of this study, a single physiotherapist and dietitian performed the prehabilitation program to prevent provider-dependent bias. Figure 1 presents a graphical overview of the implemented colorectal pathway in NS with the study’s focus section highlighted.

### Preoperative colorectal pathway

When patients were planning for elective colorectal surgery, a physiotherapist and dietitian conducted a preoperative risk assessment for postoperative complications. Patients meeting the criteria for low aerobic capacity and/or high risk of malnutrition (see Fig. 1) were advised to participate in a prehabilitation program, incorporating modalities tailored to address their impairments (Berkel et al. 2022; Molenaar et al. 2023; Franssen et al. 2022; Cederholm et al. 2019). For at least 30 days, patients performed physical exercise training and/or received dietary counseling, which were provided by a trained physiotherapist and dietitian (both with > 15 years of experience). In addition, the prehabilitation program in NS involved treating patients with low hemoglobin levels, offering alcohol- and smoking cessation interventions, and providing psychological support. However, this is already part of usual care since the implementation of the ERAS-protocol and therefore not specifically evaluated in this study. A more detailed description of the complete care pathway is provided in Appendix 1.

### Exercise modality

The physiotherapist assessed preoperative aerobic capacity using the cardiopulmonary exercise test (CPET) or modified steep ramp test (SRT), following the protocol of previous research on preoperative risk assessment (Berkel et al. 2022; Franssen et al. 2022; Bongers 2023). Patients with a low aerobic capacity, defined as an achieved work rate at peak exercise at the modified SRT ≤ 1.5 W/kg or an oxygen uptake at the ventilatory anaerobic threshold (VAT) ≤ 11 mL/kg/min at the CPET, were deemed high-risk patients and indicated for the physical exercise training modality (Berkel et al. 2022; Beijsterveld et al. 2019).

The exercise modality consisted of four components: hospital-based high-intensity interval training (HIIT), home-based HIIT and low-intensity interval training (LIIT), functional strengthening exercises, and wearing an accelerometer (Pam AM300, Pam BV, Oosterbeek, the Netherlands) (see Fig. 1 and Appendix 1). The home-based HIIT training was performed on a cycle ergometer delivered to the patient’s home (Corival Home + , Lode BV, Groningen, the Netherlands). The physiotherapist registered the delivery of the different components, the description of training variables, and the 6–20 Borg rating of perceived exertion (RPE). Adequate training adherence was defined as three HIIT sessions a week, consisting of 14 intervals of 30 s on 60% of WR_peak_ achieved at the modified SRT, followed by 60 s at 20 W (Bongers 2023).

### Nutrition modality

The dietitian evaluated the risk of malnutrition by using the Patient-Generated Subjective Global Assessment Short Form (PG-SGA SF) and applied the Global Leadership Initiative on Malnutrition (GLIM) criteria to diagnose malnutrition (Banning et al. 2020; Jager-Wittenaar and Ottery 2017). According to the GLIM-criteria, malnutrition is defined as the presence of at least one phenotypic criterion (non-volitional weight loss, low body mass index, and reduced muscle mass) and at least one etiologic criterion (reduced food intake or assimilation, and inflammation or disease burden) (Cederholm et al. 2019). Muscle mass and body composition were assessed with bioelectrical impedance analysis (BIA) (Bodygram Plus, Akern, Italy) (Goes et al. 2021). Patients with a high risk of complications due to impaired nutritional status were referred for the nutritional support modality of the prehabilitation program.

The nutrition modality consisted of counseling sessions, stimulation of protein intake, and use of the eiFIT-application. The counseling sessions aimed to optimize energy and protein intake and the timing of eating protein-rich products. If necessary, a vitamin D and leucine-enriched whey protein oral nutritional supplement (FortiFit® Powder, Nutricia) was provided. Individual protein requirements were set at 1.5–1.9 g/kg fat-free mass. Patients used the eiFIT-application or a food diary to track their protein intake (AlleyApp. 2021).

## Outcomes

### Primary outcome: implementation fidelity

**Fig. 2:**
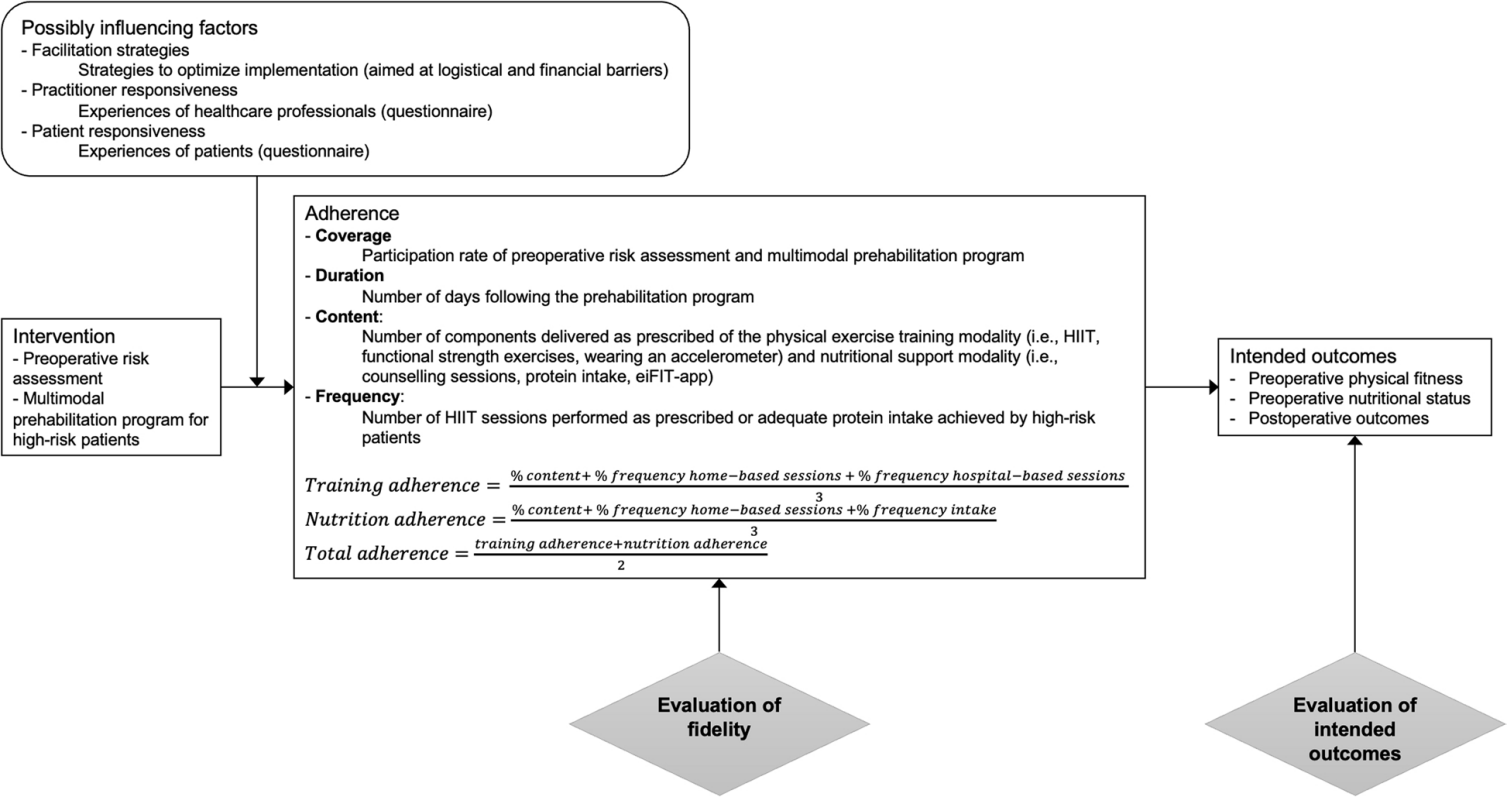
The conceptual framework of fidelity adapted to the colorectal care pathway in high-risk patients (Carroll et al. 2007)

The conceptual framework for fidelity described by Carroll et al. was used as guidance to assess the implementation fidelity of the prehabilitation program of high-risk patients (Carroll et al. 2007). The original framework has been adapted to the context of this study (Fig. 2) (Franssen et al. 2022; Pearson et al. 2020).

According to this framework, fidelity consists of adherence, the intervention components, its intended outcomes, and the potential factors influencing adherence.

### Adherence

The central aspect of the model is adherence, which can be subdivided into the following parameters: coverage, duration, content, and frequency. Coverage was defined as ‘the participation rate in the innovation by the intended audience’. It was measured by the percentage of eligible patients who were assessed and able to follow the personalized prehabilitation pathway in the study period (Pearson et al. 2020). Reasons for drop-out and non-participation were reported. Duration was measured by the number of days between assessment and surgery and should be at least 30 days (Cuijpers et al. 2022). Content was measured by the number of different components of the intervention delivered. The frequency of exercise was measured by the percentage of an adequately performed training session as prescribed. Frequency of nutrition was determined by the percentage of days in which the prescribed nutritional intake was achieved. An aggregated percentage of content and frequency was calculated to determine adherence (see Fig. 2 and Table 1). Adherence was categorized as “low” (0–60%), “moderate” (60–75%), and “high” (75–100%) (Bragstad et al. 2019).

**Table 1:** Operationalization of adherence parameters adapted to this study

	Operationalization
Coverage	$\text{Coverage}=\frac{\text{Patients assessed by the physiotherapist and dietitian }}{\text{Patients undergoing colorectal surgery in NS}}$
Duration	$\text{Duration}=\text{Date of surgery}-\text{date of preoperative assessment}$
Content	$\text{Content exercise}=\text{number of components delivered }(\text{HIIT hospital},\text{ HIIT home},$ $\text{strengthening exercises},\text{ wearing an accelerometer})$ $\text{Content nutrition}=\text{number of components delivered }(\text{counselling sessions},$ $\text{protein intake},\text{ eiFITapp})$
Frequency^a^	$\text{Frequency exercise}=\frac{\text{adequate training sessions at hospital and home}}{\text{prescribed training sessions at hospital and home}}$ $\text{Frequency nutrition}=\frac{\text{adequate counselling sessions and protein intake}}{\text{prescribed counselling sessions and protein intake}}$
Adherence	$\text{Exercise adherence}= \frac{\text{\% content}+\text{\% frequency hospital}+\text{\% frequency home}}{3}$ $\text{Nutrition adherence}=\frac{\text{\% content}+\text{\% frequency sessions}+\text{\% frequency intake}}{3}$ $\text{Total adherence}=\frac{\text{exercise adherence}+\text{nutrition adherence}}{2}$

^a^An adequately performed training session was defined as a high-intensity interval training session consisting of 14 intervals of 30 s on 60% of WR_peak_ at the modified steep ramp test followed by 60 s at 20 W or a low-intensity interval training consisting of intervals of 30 s at 30% of WR_peak_ at the modified steep ramp test, followed by 60 s at 20 W; an adequate nutritional intake was defined as a protein intake of 1.5–1.9 g/kg fat-free mass

### Secondary outcome: intended outcomes and possible influencing factors

#### Evaluation of intended outcomes

The intended outcomes of the multimodal prehabilitation program were an increase in preoperative aerobic capacity and improved preoperative nutritional status (PG-SGA SF score 0–3) (Banning et al. 2020; Weemaes et al. 2021), leading to a decrease in postoperative complications, diminished length of stay, and reduced time to in-hospital recovery of physical functioning. Preoperative aerobic capacity and nutritional status were measured before and after the prehabilitation program by the modified SRT and PG-SGA SF. The occurrence of complications was reported, and complications were categorized according to the Clavien-Dindo classification (Berkel et al. 2022; Clavien et al. 2009). Length of stay was defined as days admitted to the hospital (Berkel et al. 2022). Time to recovery of physical functioning was measured by the modified Iowa Level of Assistance Scale (mILAS). It was reported as the time in days between surgery and full in-hospital recovery of physical functioning (a mILAS score of 0) (Beijsterveld et al. 2019; Shields et al. 1995).

#### Potential influencing factors

Potential factors influencing the level of adherence explored in this study were facilitation strategies, practitioner responsiveness, and patient responsiveness. Facilitation strategies are strategies to optimize implementation aimed at barriers known from previous research, which were logistical and financial challenges (Molenaar et al. 2023; Heil et al. 2022b). The physiotherapist, dietitian, and embedded researcher documented their observations on influencing factors in a logbook. Practitioner- and patient responsiveness was defined as “engagement with the intervention.” Practitioner responsiveness was measured by a short questionnaire for the physiotherapist and dietitian based on a measurement instrument for determinants of innovation at the end of the study period (Appendix 2) (Fleuren et al. 2014). Patient responsiveness was measured by a short questionnaire for patients after finishing their prehabilitation program, based on questionnaires used in comparable studies (Appendix 3) (Franssen et al. 2022; Dronkers et al. 2010).

Clinical characteristics of participating patients following the colorectal pathway were collected for descriptive purposes (Table 2).

**Table 2:** Characteristics of low-risk and high-risk patients following the colorectal pathway

	Low-risk (*n* = 31)	High-risk (*n* = 10)
Age (years)	70 [55–73]	74 [72–78]
Sex, female	43%	90%
Living situation		
Together	75%	60%
Alone	25%	40%
Body mass index (kg/m^2^)	26 [23-29]	32 [28-34]
Smoking, no	86%	80%
Hemoglobin (mmol/L)	8.4 [7.9–8.6]	8.1 [7.9–8.4]
ASA-score		
I	14%	0%
II	75%	60%
III	11%	40%
IV	0%	0%
Charlson Comorbidity Index		
2–3	30%	10%
4–5	57%	30%
6 +	13%	60%
Time to surgery (days)	19 [14-27]	44 [35-45]
Tumor location		
Colon	75%	80%
Rectum	14%	–
No tumor	11%	20%
Surgical approach		
Laparoscopic	96%	100%
Open	4%	0%
Type of surgery		
Hemicolectomy	50%	60%
Sigmoid resection	18%	10%
Low anterior resection	4%	0%
Other	28%	30%
VO_2_ at the VAT (mL/kg/min)	12 [12-12]^d^	9 [9–9.5]^e^
Modified steep ramp test WR_peak_ (W/kg)	2.3 [2.1–2.6]^a^	1.6 [1.2–2.0]^b^
Handgrip strength (kg)	37 [30-43]	24 [22-28]
PG-SGA SF (score)	2 [0–4]	2[0–5]
Bioelectrical impedance analysis		
Fat-free mass (kg)	56 [48–65]^c^	52 [45–58]
Fat-free mass index(kg/m^2^)	19 [17-20]	19 [18-21]
Appendicular skeletal muscle mass(kg)	22 [18-25]	19 [16-22]
Length of stay (days)	3[2-4]	3 [3-11]
Postoperative complications, Yes (total group = 32%)	25%	40%
Clavien-Dindo classification (*n*)		
I	1	0
II	1	0
III	4	1
IV	1	2
V	0	1
Time to recovery of physical functioning	1[1-1]	1[1-11]
0 days	24%	11%
1 day	56%	56%
2 days	12%	11%
3 days	4%	0%
> 3 days	4%	22%

Data are presented as number of patients (%) or median [IQR] unless stated otherwise

*ASA* American Society of Anesthesiologists, *PG-SGA SF* Patient-Generated Subjective Global Assessment Short Form, *VAT* ventilatory anaerobic threshold, *VO*_*2*_ oxygen uptake, *WR*_*peak*_ work rate at peak exercise

^a^*n* = 30

^b^*n* = 8

^c^*n* = 28

^d^*n* = 1

^e^*n* = 4

### Statistical analysis

All data were analyzed using descriptive statistics. For patient characteristics, continuous data were tested for normality using Shapiro–Wilk tests and QQ-plots. Median and interquartile range (IQR) or mean and standard deviation were reported accordingly. Absolute values and percentages were given to report the fidelity of the prehabilitation program. To prevent selection bias, NS reimbursed the expenses of prehabilitation for people who otherwise could not afford it, as insurance companies did not reimburse prehabilitation in the Netherlands during the study period. Data were analyzed using R Framework 4.2.2 for macOS (version 2022, Vienna) (R Core Team. R 2022).

## Results

### Characteristics of patients in the colorectal surgery pathway

**Fig. 3:**
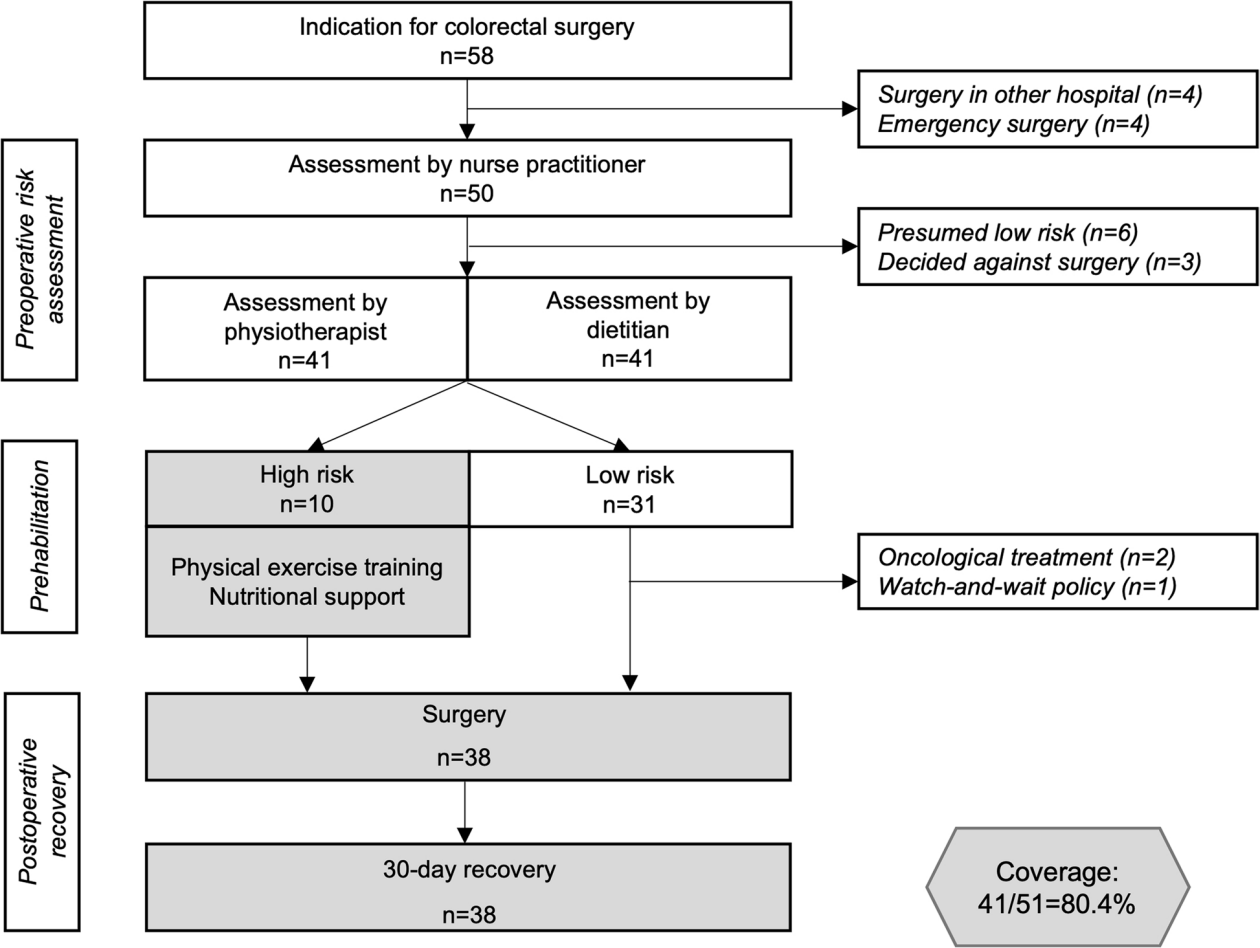
Flowchart of the colorectal surgery pathway of NS during the study period with the study’s focus section highlighted

During the study period, 58 patients started the colorectal surgery pathway, as shown in Fig. 3. Four patients underwent emergency surgery and four had surgery in a different hospital, as that hospital specialized in rectal resections. Nine patients were not referred to the physiotherapist and dietitian after assessment by the nurse practitioner, as clinical reasoning determined it unnecessary due to their low risk of complications or the patients’ personal decision to decline surgery. The physiotherapist and dietitian assessed 41 patients and the perioperative parameters of all these patients were collected. Ten (23%) of the assessed patients were classified as high-risk and, therefore, advised to follow the multimodal prehabilitation program. The clinical characteristics of the 41 patients who followed the colorectal pathway are shown in Table 2.

### Implementation fidelity

Relevant characteristics and implementation fidelity measurements of the ten high-risk patients are shown in Table 3, ordered from the highest mean adherence to the lowest. ID1 and ID9 were identified as high-risk in the assessment by the dietitian, but not according to the assessment by the physiotherapist. Consequently, the exercise modality was not indicated for them.

**Table 3:** Fidelity to the multimodal prehabilitation program followed by high-risk patients preparing for colorectal surgery in a regional hospital

					Adherence											Intended outcomes				
Patient characteristics					Duration	Content and frequency exercise modality					Content and frequency nutrition modality									
ID	CCI	DASI (METs)	SRT (W/kg)	PG-SGA SF	Intake-surgery (days)	HIIT hospital	HIIT home	Strength training	Accelerometer	Exercise adherence	Counseling sessions	Protein + vitamin supplement	eiFIT-application	Nutrition adherence	Total adherence	Aerobic capacity	Risk of malnutrition	Complications (CD)	Length of stay (days)	Functional recovery (days)
						(prescribed | done | adequate)					(prescribed | done)									
1 ♀	5	> 7	2.29	9	43	–	–	–	–		Yes (4|4)	Yes (42|42)	Yes	100%	100%	NA	NA	No	3	1
2 ♀	3	5.62	1.79	1	44	Yes (8|8|8)	Yes (8|8|8)	Yes	Yes	100%	Yes (4|4)	Yes (26|26)	Yes	100%	100%	+ 11%	Low	No	3	1
7 ♂	5	5.07	1.18	0	33	Yes (5|5|5)	Yes (7|6|5)	Yes	Yes	91%	Yes (5|4)	Yes (23|20)	Yes	89%	90%	+ 3%	Low	IVa	16	16
3 ♀	6	5.62	1.42	0	48	Yes (11|11|11)	Yes (8|8|8)	Yes	No	92%	Yes (7|7)	Yes (48|34)	No	79%	85%	+ 2%	Low	No	3	0
10♀	6	5.07	1.82	10	36	Yes (11|11|11)	Yes (6|4|4)	Yes	No	81%	Yes (4|4)	Yes (32|30)	No	87%	84%	+ 15%	Low	No	4	1
9 ♀	5	6.18	2.02	10	36	–	–	–	–		Yes (4|4)	Yes (21|11)	No	73%	73%	NA	Medium	IVa	15	2
8 ♀	8	4.64	0.86	5	53	Yes (11|11|10)	Yes (13|6|6)	Yes	No	71%	Yes (8|5)	Yes (53|47)	No	73%	72%	+ 21%	Medium	No	4	1
4 ♀	9	3.97	0.89	2	84	No	Yes (12|12|6)	Yes	No	33%	Yes (1|1)	–	No	75%	54%	+ 13%	NA	V	NA	NA
5 ♀	7	3.97	NA	4	30 + 21	No	Yes (12|3|0)	Yes	No	17%	Yes (4|4)	Yes (29|29)	No	89%	53%	NA	Low	No	3	1
6 ♀	6	3.97	NA	0	44	Yes (10|5|0)	No	Yes	No	17%	Yes (3|2)	No	No	33%	25%	NA	NA	IIIa	14	14

♀ female, ♂ male, – not indicated, *CCI* Charlson Comorbidity Index, *CD* Clavien Dindo, *HIIT* high intensity interval training, *ID* patient identification number, *PG-SGA SF* Patient-Generated Subjective Global Assessment short form, *SRT* modified steep ramp test, *WR*_*peak*_ work rate at peak exercise

*Explanation*: ID2 was in the prehabilitation program for 44 days, during which she performed hospital-based HIIT training. She had 8 prescribed sessions, performed 8 sessions, and had 8 adequate training sessions*. She should have had 4 nutritional counseling sessions and attended all 4. She received nutritional advice for protein intake, She should have followed these recommendations for 42 days and followed these recommendations for 42 days. * an adequately performed physical exercise training session was defined as a high-intensity interval training consisting of 14 intervals of 30 s at 60% of the modified SRT WR_peak_ followed by 60 s at 20 W or low-intensity interval training consisting of intervals of 30 s at 30% of the modified SRT WR_peak_, followed by 60 s at 20 W

### Adherence

The average adherence to the multimodal prehabilitation program for high-risk patients was 74%, with 63% for exercise modalities and 80% for nutrition modalities.

Seven patients were unable to use the eiFit-app and the accelerometer because of digital illiteracy or unwillingness due to its perceived burden. ID4, ID5, and ID6 had low adherence as both the content and frequency were insufficient. ID4 and ID5 were unable to perform hospital-based HIIT training due to logistical reasons, while ID6 faced difficulties performing home-based HIIT training due to a lack of internet connection.

ID5 and ID6 had low adherence at the prescribed intensity for the exercise intervention (0%) as they were unable to cycle due to their physical impairments. Based on clinical expertise, the HIIT training was adjusted to aerobic walking training in these patients. However, this type of training did not always challenge their cardiovascular system as much as high-intensity interval training, as indicated by Borg-RPE and heart rate. ID4, ID6, and ID8 did not perform half of the exercise sessions and missed some counseling sessions because they were not feeling well due to COVID-19, urinary tract infection, nausea, or dizziness.

### Evaluation of intended outcomes

Aerobic capacity improved in six patients (75%) who followed the exercise modality, deteriorated in none, and measurements were unavailable in two patients (25%). Five patients achieved a low risk of malnutrition (56%), two patients had a medium risk at the end of the prehabilitation program (22%), and measurements were unavailable in two patients (22%). Eight out of the twenty post-prehabilitation tests were missing (40%), as therapists faced unforeseen circumstances like the inability of patients to perform the tests, absence of therapists, advancements in surgeries, oversights by therapists, or unwillingness of patients to come to the hospital. When patients were unable to perform an exercise test on a cycle ergometer, the 2-min walk test (2MWT) was administered. Four patients (40%) developed complications after surgery, including two relaparotomies, one placement of a gastric tube, and one death. Median [IQR] length of stay was 3 [3-11] days and time to full in-hospital recovery of physical functioning (a mILAS score of 0) was 1 day [1-11].

### Potential influencing factors

Potential influencing factors investigated in this study were facilitation strategies, practitioner responsiveness, and patient responsiveness. Facilitation strategies include flexible appointment scheduling by the physiotherapist and the dietician, which can help overcome patients’ logistical challenges. Another facilitation strategy to address logistical challenges was offering therapy at home and in the hospital. A facilitation strategy to address financial barriers was the choice of NS to reimburse the expenses of prehabilitation for people who otherwise could not afford it. The practitioner responsiveness was good (Appendix 2), especially for the outcome expectations. Patient responsiveness was also good, as all patients indicated high motivation for the program (Table 4).

**Table 4:** Patient responsiveness: questionnaire results

ID	1	2	3	4	5	6	7	8	9	10
1. The aim of the prehabilitation program was clear to me	+ / −	+ +	−	+ +	+ +	+ +	+ +	+ +	+ +	+ +
2. The experienced exertion during the exercises was high	NA	+ / −	+ / −	+	+	−	+ +	+ +	NA	+ / −
3. The exercises were useful	NA	+ +	+ +	+ +	+ +	+ +	+ +	+ +	NA	+ +
4. The nutritional support was useful	+ +	+ +	+ +	+ +	+ +	+ +	+ +	+ +	+ / −	+ +
5. The exercises were easy to maintain	NA	+ / −	+ +	+	+ +	+ +	+ +	−	NA	+
6. The nutritional recommendations were easy to maintain	+	+ +	+ +	+	+ +	+ +	+ +	+ +	+	+
7. I was motivated for the prehabilitation program	+ +	+ +	+	+	+ +	+ +	+ +	+ +	+ +	+ +
8. I think the prehabilitation program prepared me well for surgery	+ +	+	+ +	+	+ +	+ +	+ +	+	+ / −	+ +

+ + strongly agree, + agree, + / − neutral, − disagree, − − strongly disagree

## Discussion

To the best of our knowledge, this is the first study that investigated the implementation fidelity of an evidence-based multimodal prehabilitation program for high-risk patients undergoing elective colorectal surgery and explored its intended outcomes. The results of this cohort study with multiple case analyses suggest that the implementation fidelity was moderate, as adherence varied between good (*n* = 5), moderate (*n* = 2), and low (*n* = 3). Patients with low adherence struggled to execute the different components (content) and frequency of mainly the physical exercise training modality, due to multiple physical and cognitive impairments. Aerobic capacity and nutritional status improved preoperatively in all six patients for whom evaluation was possible; however, evaluation was not feasible in four patients. Four out of the 10 high-risk patients developed severe complications after surgery (Clavien-Dindo IIIa-V).

The adherence rates were moderate to high for most patients, which is a favorable outcome given the complexity of the multimodal intervention for high-risk patients in a real-world clinical setting. Several factors influencing the program could be identified, such as the patient’s motivation for the program and the facilitation strategies to address logistical and financial barriers. In this study, the adherence to nutritional modalities was higher than the adherence to the exercise modalities. The personalized nature of the nutritional intervention, combined with the dietitian’s counseling sessions during a patient’s physical exercise training session at the hospital, might have contributed to this outcome. Adherence to the nutritional modalities was rarely described in previous studies. A comparable study investigating a tele-prehabilitation program achieved a frequency of 91% and intensity of 84% for the exercise program (Franssen et al. 2022). This is higher than we achieved in our home-based components. Unfortunately, only three patients were able to carry out all the digital home-based components. This suggests that an intervention only containing digital home-based components might not suit this population. Blended care could be considered an appropriate option, since the possibility of providing both home-based training and hospital-based training might contribute to flexibility and therefore higher adherence to the prehabilitation program.

Adherence varied between patients (25–100%), with remarkably low adherence (< 55%) in three of these patients. These three patients had impairments, such as comorbidities, low functional status, cognitive decline, and lack of social support. Efforts were taken to adapt the intervention as much as possible for the individual patients, for example, switching to only home-based or only hospital-based training and altering training on the cycle ergometer to walking training. It required flexibility and clinical expertise by the healthcare professionals to adjust the program to the patients’ abilities and support them during their prehabilitation program. The questionnaire for practitioner responsiveness supported this inference, showing that the physiotherapist and dietitian experience the prehabilitation program as time-consuming, and they are hesitant about the ability to help every patient and the visibility of the effects. In previous research, no suggestions were made for adapting the assessment and intervention to the needs of patients with impairments awaiting surgery, even though this is essential for the intervention’s success (Carroll et al. 2007). Clinical trials for prehabilitation programs often exclude patients who cannot perform cycling tests or prescribed exercises (Berkel et al. 2022; Molenaar et al. 2023). Conversely, it can be assumed that these patients are more prone to negative postoperative outcomes based on their low functional status. Scientific recommendations for preoperative optimalization for patients at high risk with multiple impairments are currently unavailable, to the best of our knowledge.

The intended outcomes (effects comparable to those found in previous RCTs) were realized to a certain extent; while the improvement in preoperative aerobic capacity fell short, a decreased risk of malnutrition and favorable postoperative outcomes were achieved in 6 out of 10 patients. The changes in preoperative aerobic capacity were less than those previously reported in two RCTs with comparable prehabilitation programs (Berkel et al. 2022; Molenaar et al. 2023). A recent clinical trial also found variations in outcomes after prehabilitation, which were explained by “non-responders” (Berkel et al. 2022). That study acknowledged the challenge of improving aerobic capacity in all high-risk patients. Furthermore, the reduction in the risk of malnutrition was comparable to the improvement found in a recent RCT (Molenaar et al. 2023). Length of stay and time to recovery of physical functioning was comparable to earlier research (Berkel et al. 2022; Molenaar et al. 2023; Thomas et al. 2019). The complication rate of the total group was 32%, which is lower or similar to the complication rate in intervention groups of earlier RCTs (Molenaar et al. 2023; Thomas et al. 2019). However, these RCTs included both high-risk and low-risk patients and provided them all with a multimodal prehabilitation program of four weeks. The evaluation of intended outcomes should be treated with caution due to the small sample size, and the potential favorable outcomes should be investigated by a full-scale study.

This study reveals the real-life context involving patients with multiple impairments, highlighting the importance of personalized preventive care. For such patients, prehabilitation becomes crucial for optimizing their condition before surgery. However, since these types of patients have been excluded from most previous RCTs, the one-size-fits-all recommendations often cannot be applied to this subgroup. Success in real-life prehabilitation hinges on personalized interventions, which require healthcare professionals to adapt and ensure flexibility in the preoperative pathway.

The strength of this study is the observational nature of real-life practice, which provides a solid impression of the clinical practice of prehabilitation and its challenges. An embedded scientist and physiotherapist actively participated in the real-life practice and facilitated the process. Data were gathered through desk research, incorporating the perspectives of patients, practitioners, and observations. In situations where implementation challenges arose (e.g., difficulties administering measurement tools or delivering therapy to specific patients), the embedded researcher provided clinical guidance to adapt strategies in real time. While such involvement in the implementation process introduces a potential risk of bias, the role of a reflective, embedded scientist remains vital to achieving sustainable science (Wittmayer and Schäpke 2014). Seeking to mitigate bias, the embedded researcher triangulated data from multiple resources and engaged in direct consultations with healthcare professionals. Open discussions with the physiotherapist and dietitian encouraged honest feedback, enabling a more accurate assessment of the implementation process. These measures were designed to minimize bias and uphold the integrity and reliability of the research findings. The lack of exclusion criteria makes the results relevant for other healthcare settings and professionals in similar practices. This broader inclusivity strengthens the external validity and practical implications of the study.

The main limitation of this study is the small sample size and therefore the inability to perform statistical analyses to identify the most important components and effects of the prehabilitation program (Carroll et al. 2007). With such a sample size, a mixed-methods process evaluation might have been more appropriate to gain a deeper understanding of the process. However, descriptive variables and detailed fidelity reporting, using a commonly employed framework, provide valuable insights into the existing literature on barriers and facilitators for the implementation of prehabilitation (Ginsburg et al. 2021).

In future research, adaptations to the current scientific recommendations for high-risk patients should be studied and validated. It is recommended to perform mixed-methods process evaluations of prehabilitation in real-life contexts. An evaluation using the quadruple aim framework is advised, assessing not only outcomes but also cost-effectiveness, patient perspectives, and healthcare professional perspectives (Sikka et al. 2015). Research should include a sufficient sample size and component analysis to identify key components that explain the variance in practices and associated outcomes within real-life practice. In real-world clinical settings, physiotherapists and dietitians should pay attention to frail patients by being flexible in the content of their personalized prehabilitation program.

## Conclusion

The implementation fidelity of an evidence-based multimodal prehabilitation program for high-risk patients preparing for colorectal surgery in real-life practice was moderate, as adherence was low in patients with multiple impairments. For healthcare professionals, it is recommended to pay attention to high-risk patients with multiple impairments and further personalize the prehabilitation program. More knowledge about identifying and treating high-risk patients is needed to provide evidence-based recommendations and increase effectiveness.

## Figures and Tables

**Figure:**
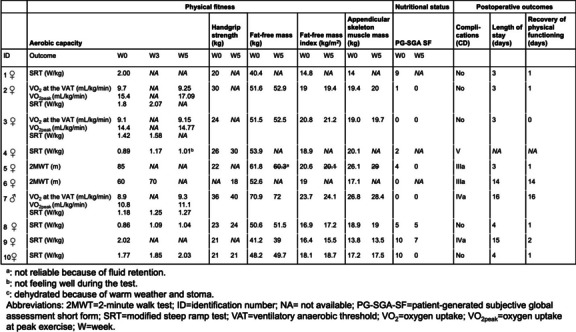
Preoperative physical fitness and nutritional status

## Data Availability

Data are pseudonymized and stored in a secured environment. The data that support the findings of this study are available from Hanzehogeschool Groningen, but restrictions apply to the availability of these data, which were used under license for the current study, and so are not publicly available. Data are however available from the authors upon reasonable request and with permission Hanzehogeschool Groningen.

## Appendix 1

### Colorectal pathway of Nij Smellinghe

NS is a regional hospital with 339 beds in the northern part of The Netherlands, in which approximately 150 colorectal surgeries are conducted each year. All patients are screened by a nurse practitioner, who also serves as a case manager. In the current study period, there were two nurse practitioners, one dietitian, and one physiotherapist involved. There are five different surgeons who perform the surgeries, of which most are laparoscopic. Patients are all treated according to the ERAS protocol, in which all nurses and physiotherapists are trained.

The physiotherapeutic assessment of NS is based on the study of Berkel, et al. (2022). Patients with a score £ 7 metabolic equivalents of task (METs) at the Duke Activity Status Index (DASI) performed a (CPET). Patients with a score of > 7 METs at the DASI performed a modified Steep Ramp Test. The tests are admitted on calibrated cycle ergometers (Lode Corival, Lode BV, Groningen, The Netherlands). Patients with an oxygen uptake (VO_2_) at the ventilatory anaerobic threshold (VAT) £ 11 mL/kg/min at the CPET or a work rate at peak exercise (WR_peak_) £ 1.5 W/kg at the modified SRT are considered to be at high risk of postoperative complications and therefore advised to follow the preoperative physical exercise training program of NS.

The preoperative physical exercise training program of NS consists of a hospital-based training session two times a week and home-based exercise five times a week. Hospital-based exercise consist of high-intensity interval training (HIIT) and functional strength exercises. The patient is asked frequently for their 6–20 Borg rating of perceived exertion (RPE) to objectively monitor the perceived training intensity. When patients score less than Borg RPE 13–15 in the high-intensity intervals or show no progress in their modified SRT performance at three weeks, the training intensity will be increased, taking into account patients’ experiences and preferences Franssen, et al. (2022). In this way, the concept of titration is applied to make an individually tailored intervention to avoid non-response to the intervention Beijsterveld, et al. (2021). The home-based exercise consists of one HIIT session, four low-intensity interval training (LIIT) sessions, and functional strength exercises. The HIIT training consisted of intervals of 30 s at 60% of the SRT WR_peak_ followed by 60 s at 20 W Bongers (2023). The LIIT training consisted of intervals of 30 s at 30% of SRT WR_peak_ followed by 60 s at 20 W. The HIIT and LIIT were performed on a stationary cycle ergometer (Lode Corival Home+, Lode BV, Groningen, The Netherlands) that was delivered at the patient’s home. This cycle ergometer was introduced and installed during a home visit by the physiotherapist. Patients are advised to work towards a Borg RPE £ 11 during their home exercises Franssen, et al. (2022). The training parameters and completed Borg scores are displayed in an online dashboard to which the physiotherapist has access. The physiotherapist checks once a week if the home-based training sessions are performed properly. Patients also wore an accelerometer to monitor physical activity behavior. The accelerometer (Pam AM300, Pam BV, Oosterbeek, The Netherlands) will only be used to check if patients meet the norm for physical activity.

The dietitian assesses the risk for malnutrition by using the patient-generated subjective global assessment short form (PG-SGA SF), after which the nutritional status is assessed with the bioelectrical impedance analysis (BIA) (Bodygram Plus, Akern, Italy). These are validated instruments used by dietitians for the assessment of malnutrition and monitoring of interventions Cederholm (2019), Goes (2021), Nwosu (2019). Patients with a high risk for malnutrition, according to the Global Leadership Initiative on Malnutrition (GLIM) criteria, were indicated as high-risk patients and were advised to follow the nutrition program of NS Asseldonk (2022).

The nutritional support of NS consists of stimulating energy and protein intake, optimization of timing protein intake, and, if necessary, additional protein or vitamin supplements. Required protein intake was 1.2–1.5 g/kg body mass. The nutrition program is individualized and adjusted to a patient’s needs, as indicated by the assessment. The intervention will be evaluated and modified if needed in face-to-face or telephonic contact sessions. Protein intake and compliance with nutritional advice will be monitored by the eiFIT app or a personalized diary. The frequency of sessions will be adjusted to a patient’s preferences and the dietitian’s assessment.

## Appendix 2

**Table 5:** Practitioner responsiveness

	Strongly disagree	Disagree	Neutral	Agree	Strongly agree
The innovation provides clear guidance on the sequence of activities to be performed.				PTDT	
The innovation is based on accurate knowledge.				DT	PT
The innovation provides all the necessary information and materials to work with effectively				PTDT	
The innovation is too complex for me to use.	PTDT				
The innovation aligns well with my usual way of working.				PTDT	
I can clearly see the effects of using the innovation.			DT	PT	
I find the innovation suitable for my patients.				PTDT	
Benefit: The innovation enables me to assist all patients in a suitable manner.			PT		DT
Drawback: The innovation requires a significant time investment from me.				DT	PT
I consider it important to use the innovation to improve the patient's fitness.					PTDT
I expect that the innovation will effectively achieve the goal of improving the patient's fitness.					PTDT

## Abbreviations

2MWT: 2-Minute walk test
ASA: American Society of Anesthesiologists
ASMM: Appendicular skeleton muscle mass
BIVA: Bioelectrical impedance vector analysis
BMI: Body mass index
CCI: Age-adjusted Charlson Comorbidity Index
CPET: Cardiopulmonary exercise test
DASI: Duke Activity Status Index
DT: Dietitian
ERAS: Enhanced recovery after surgery
FFM: Fat-free mass
FFMI: Fat-free mass index
HGS: Handgrip strength
MET: Metabolic Equivalent of Task
mILAS: Modified Iowa Level of Assistance scale
NP: Nurse practitioner
NS: Nij Smellinghe (regional hospital in The Netherlands)
PG-SGA-SF: Patient-generated subjective global assessment short form
PT: Physical therapist
RCT: Randomized controlled trial
RPE: Rating of perceived exertion
SRT: Steep ramp test
STROBE: STrengthening the Reporting of Observational Studies in Epidemiology

## References

[CR1] AlleyApp. EiFit application. Amsterdam; 2021.

[CR2] Banning LBD, ter Beek L, El Moumni M, Visser L, Zeebregts CJ, Jager-Wittenaar H, et al. Vascular surgery patients at risk for malnutrition are at an increased risk of developing postoperative complications. Ann Vasc Surg. 2020;64:213–20. 31634605 10.1016/j.avsg.2019.10.037

[CR3] Bauer MS, Kirchner J. Implementation science: What is it and why should I care? Psychiatry Res. 2020;283:112376. 31036287 10.1016/j.psychres.2019.04.025

[CR4] Berkel AEM, Bongers BC, Kotte H, Weltevreden P, de Jongh FHC, Eijsvogel MMM, et al. Effects of community-based exercise prehabilitation for patients scheduled for colorectal surgery with high risk for postoperative complications: results of a randomized clinical trial. Ann Surg. 2022 Feb [cited 2022 Sep 16];275(2):e299–306. Available from: https://journals-lww-com.nlhhg.idm.oclc.org/ejanaesthesiology/Fulltext/2019/12000/Prehabilitation_before_major_intra_abdominal.6.aspx 10.1097/SLA.0000000000004702 PMC8746915 33443905

[CR5] Bongers BC. Steep ramp test protocol for preoperative risk assessment and short-term high-intensity interval training to evaluate, improve, and monitor cardiorespiratory fitness in surgical oncology. J Surg Oncol. 2023;127(5):891–5. 36621860 10.1002/jso.27201

[CR6] Bragstad LK, Bronken BA, Sveen U, Hjelle EG, Kitzmüller G, Martinsen R, et al. Implementation fidelity in a complex intervention promoting psychosocial well-being following stroke: An explanatory sequential mixed methods study. BMC Med Res Methodol. 2019;19(1):59. 10.1186/s12874-019-0694-z PMC6419826 30876403

[CR7] Bussemaker J, KJ, de LM. Zonder context geen bewijs. In: van Dijken, P, Barnhoorn, P, Geurts, J (eds) Professionaliteit in de zorg Bohn Stafleu van Loghum, Houten. 2021;

[CR8] Carli F, Silver JK, Feldman LS, McKee A, Gilman S, Gillis C, et al. Surgical prehabilitation in patients with cancer: state-of-the-science and recommendations for future research from a panel of subject matter experts. Vol. 28, Physical Medicine and Rehabilitation Clinics of North America. W.B. Saunders; 2017. p. 49–64. 10.1016/j.pmr.2016.09.002 27913000

[CR9] Carroll C, Patterson M, Wood S, Booth A, Rick J, Balain S. A conceptual framework for implementation fidelity. Implement Sci. 2007;2(1):40. 18053122 10.1186/1748-5908-2-40 PMC2213686

[CR10] Cederholm T, Jensen GL, Correia MITD, Gonzalez MC, Fukushima R, Higashiguchi T, et al. GLIM criteria for the diagnosis of malnutrition – a consensus report from the global clinical nutrition community. Clin Nutr. 2019;38(1):1–9. 30181091 10.1016/j.clnu.2018.08.002

[CR11] Clavien PA, Barkun J, de Oliveira ML, Vauthey JN, Dindo D, Schulick RD, et al. The Clavien-Dindo Classification of surgical complications. Ann Surg. 2009;250(2):187–96. 19638912 10.1097/SLA.0b013e3181b13ca2

[CR12] Cortés-Guiral D, Mohamed F, Glehen O, Passot G. Prehabilitation of patients undergoing cytoreductive surgery (CRS) and hyperthermic intraperitoneal chemotherapy (HIPEC) for peritoneal malignancy. Eur J Surg Oncol. 2021;47(1):60–4. 32063398 10.1016/j.ejso.2020.01.032

[CR13] Cuijpers ACM, Linskens FG, Bongers BC, Stassen LPS, Lubbers T, van Meeteren NLU. Quality and clinical generalizability of feasibility outcomes in exercise prehabilitation before colorectal cancer surgery – A systematic review. Vol. 48, Eur J Surg Oncol. W.B. Saunders Ltd; 2022. p. 1483–97. 10.1016/j.ejso.2022.04.012 35491361

[CR14] de Klerk M, van Dalen DH, Nahar-van Venrooij LMW, Meijerink WJHJ, Verdaasdonk EGG. A multimodal prehabilitation program in high-risk patients undergoing elective resection for colorectal cancer: A retrospective cohort study. Eur J Surg Oncol. 2021;47(11):2849–56. 34103244 10.1016/j.ejso.2021.05.033

[CR15] Dronkers J, Lamberts H, Reutelingsperger I, Naber R, Dronkers-Landman C, Veldman A, et al. Preoperative therapeutic programme for elderly patients scheduled for elective abdominal oncological surgery: a randomized controlled pilot study. Clin Rehabil. 2010;24(7):614–22. 20530651 10.1177/0269215509358941

[CR16] Durrand J, Singh SJ, Danjoux G. Prehabilitation. Clin Med. 2019;19(6):458–64. 10.7861/clinmed.2019-0257 PMC6899232 31732585

[CR17] Fleuren MAH, Paulussen TGWM, Van Dommelen P, Van Buuren S. Towards a measurement instrument for determinants of innovations. Int J Qual Health Care. 2014;26(5):501–10. 24951511 10.1093/intqhc/mzu060 PMC4195468

[CR18] Franssen RFW, Bongers BC, Vogelaar FJ, Janssen-Heijnen MLG. Feasibility of a tele-prehabilitation program in high-risk patients with colon or rectal cancer undergoing elective surgery: a feasibility study. Perioper Med. 2022;11(1):28. 10.1186/s13741-022-00260-5 PMC9313601 35879732

[CR19] Ginsburg LR, Hoben M, Easterbrook A, Anderson RA, Estabrooks CA, Norton PG. Fidelity is not easy! Challenges and guidelines for assessing fidelity in complex interventions. Trials. 2021;22(1):372. 34051830 10.1186/s13063-021-05322-5 PMC8164256

[CR20] Goes AC, Santos MA, Oliveira R de S, Oliveira J de S, Roriz AKC, de Oliveira CC. The use of bioelectrical impedance vector analysis for a nutritional evaluation of older adults in the community. Exp Gerontol. 2021;147:111276. 10.1016/j.exger.2021.111276 33571661

[CR21] Greco M, Capretti G, Beretta L, Gemma M, Pecorelli N, Braga M. Enhanced recovery program in colorectal surgery: a meta-analysis of randomized controlled trials. World J Surg. 2014;38(6):1531–41. 24368573 10.1007/s00268-013-2416-8

[CR22] Heil TC, Driessen EJM, Argillander TE, Melis RJF, Maas HAAM, Olde Rikkert MGM, et al. Implementation of prehabilitation in colorectal cancer surgery: qualitative research on how to strengthen facilitators and overcome barriers. Support Care Cancer. 2022b;30(9):7373–86. 35610321 10.1007/s00520-022-07144-w PMC9130002

[CR23] Heil TC, Verdaasdonk EGG, Maas HAAM, van Munster BC, Rikkert MGMO, de Wilt JHW, et al. Improved postoperative outcomes after prehabilitation for colorectal cancer surgery in older patients: an emulated target trial. Ann Surg Oncol. 2022 Oct 5; Available from: https://link.springer.com/10.1245/s10434-022-12623-9 10.1245/s10434-022-12623-9 PMC9533971 36197561

[CR24] Jager-Wittenaar H, Ottery FD. Assessing nutritional status in cancer. Curr Opin Clin Nutr Metab Care. 2017;20(5):322–9. 28562490 10.1097/MCO.0000000000000389

[CR25] Khuri SF, Henderson WG, DePalma RG, Mosca C, Healey NA, Kumbhani DJ. Determinants of long-term survival after major surgery and the adverse effect of postoperative complications. Ann Surg. 2005;242(3):326–43. 16135919 10.1097/01.sla.0000179621.33268.83 PMC1357741

[CR26] Lambert JE, Hayes LD, Keegan TJ, Subar DA, Gaffney CJ. The impact of prehabilitation on patient outcomes in hepatobiliary, colorectal, and upper gastrointestinal cancer surgery. Ann Surg. 2021;274(1):70–7. 33201129 10.1097/SLA.0000000000004527

[CR27] Mayo NE, Feldman L, Scott S, Zavorsky G, Kim DJ, Charlebois P, et al. Impact of preoperative change in physical function on postoperative recovery: Argument supporting prehabilitation for colorectal surgery. Surgery. 2011;150(3):505–14. 21878237 10.1016/j.surg.2011.07.045

[CR28] Molenaar CJL, Minnella EM, Coca-Martinez M, ten Cate DWG, Regis M, Awasthi R, et al. Effect of multimodal prehabilitation on reducing postoperative complications and enhancing functional capacity following colorectal cancer surgery. JAMA Surg. 2023; 10.1001/jamasurg.2023.0198 PMC10061316 36988937

[CR29] Molenaar CJL, Reudink M, Sabajo CR, Janssen L, Roumen RMH, Klaase JM, et al. Prehabilitation for patients with colorectal cancer: a snapshot of current daily practice in Dutch hospitals. Perioperative Medicine. 2023;12(1):15. 37158927 10.1186/s13741-023-00299-y PMC10165784

[CR30] Moran J, Guinan E, McCormick P, Larkin J, Mockler D, Hussey J, et al. The ability of prehabilitation to influence postoperative outcome after intra-abdominal operation: A systematic review and meta-analysis. Surgery. 2016;160(5):1189–201. 27397681 10.1016/j.surg.2016.05.014

[CR31] Morris ZS, Wooding S, Grant J. The answer is 17 years, what is the question: understanding time lags in translational research. J R Soc Med. 2011;104(12):510–20. 22179294 10.1258/jrsm.2011.110180 PMC3241518

[CR32] Nwosu AC, Mayland CR, Mason S, Cox TF, Varro A, Stanley S, et al. Bioelectrical impedance vector analysis (BIVA) as a method to compare body composition differences according to cancer stage and type. Clin Nutr ESPEN. 2019;30:59–66. 30904230 10.1016/j.clnesp.2019.02.006

[CR33] Pearson N, Naylor PJ, Ashe MC, Fernandez M, Yoong SL, Wolfenden L. Guidance for conducting feasibility and pilot studies for implementation trials. Pilot Feasibility Stud. 2020;6(1):167. 10.1186/s40814-020-00634-w PMC7603668 33292770

[CR34] Proctor E, Silmere H, Raghavan R, Hovmand P, Aarons G, Bunger A, et al. Outcomes for implementation research: conceptual distinctions, measurement challenges, and research agenda. Adm Policy Ment Health. 2011;38(2):65–76. 20957426 10.1007/s10488-010-0319-7 PMC3068522

[CR35] R Core Team. R: A language and environment for statistical computing. Vienna: R Foundation for Statistical Computing; 2022.

[CR36] Rogers L, De Brún A, McAuliffe E. Defining and assessing context in healthcare implementation studies: a systematic review. BMC Health Serv Res. 2020;20(1):591. 10.1186/s12913-020-05212-7 PMC7322847 32600396

[CR37] Sackett DL, Rosenberg WMC, Gray JAM, Haynes RB, Richardson WS. Evidence based medicine: what it is and what it isn’t. BMJ. 1996;312(7023):71–2. 8555924 10.1136/bmj.312.7023.71 PMC2349778

[CR38] Shields RK, Enloe LJ, Evans RE, Smith KB, Steckel SD. Reliability, validity, and responsiveness of functional tests in patients with total joint replacement. Phys Ther. 1995;75(3):169–76. 7870749 10.1093/ptj/75.3.169

[CR39] Sikka R, Morath JM, Leape L. The Quadruple Aim: care, health, cost and meaning in work. BMJ Qual Saf. 2015;24(10):608–10. 26038586 10.1136/bmjqs-2015-004160

[CR40] Thomas G, Tahir MR, Bongers BC, Kallen VL, Slooter GD, van Meeteren NL. Prehabilitation before major intra-abdominal cancer surgery. Eur J Anaesthesiol. 2019;36(12):933–45. 31188152 10.1097/EJA.0000000000001030 PMC6855314

[CR41] van Asseldonk M, Beijer S, Boschma T, Breedveld-Peters J, Van Erven C, Holverda M, Kohlen M, Van Lienen G, Remijnse W, Witting M. Leidraad diëtetiek Prehabilitatie. NVD. 2022.

[CR42] Van Beijsterveld CA, Bongers BC, Den Dulk M, Van Kuijk SMJ, Dejong KCH, Van Meeteren NLU. The association between preoperative physical functioning and short-term postoperative outcomes: a cohort study of patients undergoing elective hepatic resection. HPB. 2019;21(10):1362–70. 30926327 10.1016/j.hpb.2019.02.009

[CR43] van Beijsterveld CA, Bongers BC, den Dulk M, Dejong CH, van Meeteren NL. Personalized community-based prehabilitation for a high-risk surgical patient opting for pylorus-preserving pancreaticoduodenectomy: a case report. Physiother Theory Pract. 2021;37(12):1497–509. 10.1080/09593985.2019.1709233 32013652

[CR44] van der Sluis G, Goldbohm RA, Bimmel R, Galindo Garre F, Elings J, Hoogeboom TJ, et al. What augmented physical activity and empowerment can bring to patients receiving total knee replacement: content, implementation, and comparative effectiveness of a new function-tailored care pathway in a routine care setting. Biomed Res Int. 2015;2015:1–8. 10.1155/2015/745864 PMC4415657 25961038

[CR45] van Rooijen S, Carli F, Dalton S, Thomas G, Bojesen R, Le Guen M, et al. Multimodal prehabilitation in colorectal cancer patients to improve functional capacity and reduce postoperative complications: the first international randomized controlled trial for multimodal prehabilitation. BMC Cancer. 2019a;19(1):98. 30670009 10.1186/s12885-018-5232-6 PMC6341758

[CR46] Van Rooijen SJ, Molenaar CJL, Schep G, Van Lieshout RHMA, Beijer S, Dubbers R, et al. Making patients fit for surgery: introducing a four pillar multimodal prehabilitation program in colorectal cancer. Am J Phys Med Rehabil. 2019b;98(10):888–96. 31090551 10.1097/PHM.0000000000001221

[CR47] Vindrola-Padros C, Pape T, Utley M, Fulop NJ. The role of embedded research in quality improvement: a narrative review. BMJ Qual Saf. 2017;26(1):70–80. 27129492 10.1136/bmjqs-2015-004877 PMC5256405

[CR48] von Elm E, Altman DG, Egger M, Pocock SJ, Gøtzsche PC, Vandenbroucke JP. Strengthening the reporting of observational studies in epidemiology (STROBE) statement: guidelines for reporting observational studies. BMJ. 2007;335(7624):806–8. 17947786 10.1136/bmj.39335.541782.AD PMC2034723

[CR49] Wittmayer JM, Schäpke N. Action, research and participation: roles of researchers in sustainability transitions. Sustain Sci. 2014;9(4):483–96.

[CR50] Weemaes ATR, Beelen M, Bongers BC, Weijenberg MP, Lenssen AF. Criterion Validity and Responsiveness of the Steep Ramp Test to Evaluate Aerobic Capacity in Survivors of Cancer Participating in a Supervised Exercise Rehabilitation Program. Arch Phys Med Rehabil. 2021;102(11):2150–6. 34023324 10.1016/j.apmr.2021.04.016

